# SOX9 Triggers Different Epithelial to Mesenchymal Transition States to Promote Pancreatic Cancer Progression

**DOI:** 10.3390/cancers14040916

**Published:** 2022-02-12

**Authors:** Estefania Carrasco-Garcia, Lidia Lopez, Veronica Moncho-Amor, Fernando Carazo, Paula Aldaz, Manuel Collado, Donald Bell, Ayman Gaafar, Eva Karamitopoulou, Alexandar Tzankov, Manuel Hidalgo, Ángel Rubio, Manuel Serrano, Charles H. Lawrie, Robin Lovell-Badge, Ander Matheu

**Affiliations:** 1 Cellular Oncology Group, Biodonostia Health Research Institute, 20014 San Sebastian, Spain; tslab.granada@hotmail.com (L.L.); veronica.moncho@biodonostia.org (V.M.-A.); paula.aldaz.donamaria@navarra.es (P.A.); 2 CIBER de Fragilidad y Envejecimiento Saludable (CIBERfes), 28029 Madrid, Spain; 3 The Francis Crick Institute, London NW1 1AT, UK; donald.bell@crick.ac.uk (D.B.); robin.lovell-badge@crick.ac.uk (R.L.-B.); 4 School of Engineering, University of Navarra, 20009 San Sebastian, Spain; fcarazo@tecnun.es (F.C.); arubio@tecnun.es (Á.R.); 5 Health Research Institute of Santiago de Compostela (IDIS), Xerencia de Xestión Integrada de Santiago (XXIS/SERGAS), 15706 Santiago de Compostela, Spain; manuel.collado.rodriguez@sergas.es; 6 Department of Pathology, Cruces University Hospital, 48903 Barakaldo, Spain; ayman.gaafareleraky@osakidetza.eus; 7 Institute of Pathology, University of Bern, 3012 Bern, Switzerland; eva.diamantis@pathology.unibe.ch; 8 Institute of Pathology, University Hospital Basel, 4056 Basel, Switzerland; alexandar.tzankov@usb.ch; 9 Spanish National Cancer Research Centre (CNIO), 28029 Madrid, Spain; mah4006@med.cornell.edu; 10 New York-Presbyterian Hospital/Weill Cornell Medical Center, New York, NY 10065, USA; 11 Institute for Research in Biomedicine (IRB Barcelona), Barcelona Institute of Science and Technology (BIST), 08028 Barcelona, Spain; manuel.serrano@irbbarcelona.org; 12 Catalan Institution for Research and Advanced Studies (ICREA), 08010 Barcelona, Spain; 13 Molecular Oncology Group, Biodonostia Institute, 20014 San Sebastian, Spain; charles.lawrie@biodonostia.org; 14 IKERBASQUE, Basque Foundation for Science, 48009 Bilbao, Spain

**Keywords:** SOX9, pancreatic cancer, plasticity, metastasis, EMT, chemoresistance

## Abstract

**Simple Summary:**

Pancreatic cancers are lethal types of cancer. A majority of patients progress to an advanced and metastatic disease, which remains a major clinical problem. Therefore, it is crucial to identify critical regulators to help predict the disease progression and to develop more efficacious therapeutic approaches. In this work we found that an increased expression of the developmental factor SOX9 is associated with metastasis, a poor prognosis and resistance to therapy in pancreatic ductal adenocarcinoma patients and in cell cultures. We also found that this effect is at least in part due to the ability of SOX9 to regulate the activity of stem cell factors, such as BMI1, in addition to those involved in EMT and metastasis.

**Abstract:**

Background: Pancreatic ductal adenocarcinoma (PDAC) is one of the most lethal cancers mainly due to spatial obstacles to complete resection, early metastasis and therapy resistance. The molecular events accompanying PDAC progression remain poorly understood. SOX9 is required for maintaining the pancreatic ductal identity and it is involved in the initiation of pancreatic cancer. In addition, SOX9 is a transcription factor linked to stem cell activity and is commonly overexpressed in solid cancers. It cooperates with Snail/Slug to induce epithelial-mesenchymal transition (EMT) during neural development and in diseases such as organ fibrosis or different types of cancer. Methods: We investigated the roles of SOX9 in pancreatic tumor cell plasticity, metastatic dissemination and chemoresistance using pancreatic cancer cell lines as well as mouse embryo fibroblasts. In addition, we characterized the clinical relevance of SOX9 in pancreatic cancer using human biopsies. Results: Gain- and loss-of-function of SOX9 in PDAC cells revealed that high levels of SOX9 increased migration and invasion, and promoted EMT and metastatic dissemination, whilst SOX9 silencing resulted in metastasis inhibition, along with a phenotypic reversion to epithelial features and loss of stemness potential. In both contexts, EMT factors were not altered. Moreover, high levels of SOX9 promoted resistance to gemcitabine. In contrast, overexpression of SOX9 was sufficient to promote metastatic potential in *K-Ras* transformed MEFs, triggering EMT associated with Snail/Slug activity. In clinical samples, SOX9 expression was analyzed in 198 PDAC cases by immunohistochemistry and in 53 patient derived xenografts (PDXs). SOX9 was overexpressed in primary adenocarcinomas and particularly in metastases. Notably, SOX9 expression correlated with high vimentin and low E-cadherin expression. Conclusions: Our results indicate that SOX9 facilitates PDAC progression and metastasis by triggering stemness and EMT.

## 1. Introduction

Metastasis is a complex process by which cancer cells spread from the primary tumor and colonize distant tissues in the body. The process takes place through a sequence of steps including invasion across the surrounding extracellular matrix, intravasation and dissemination into the systemic circulation, and the extravasation and colonization of distant organs. The first steps of the process have been linked to the activation of the epithelial-mesenchymal transition (EMT), which confers to cancer cells mesenchymal characteristics that allow their dissemination from the primary tumor. In contrast, the “engraftment” of cancer cells in distant organs has been associated with the reverse process (i.e., mesenchymal-epithelial transition or MET) [1,2]. In EMT, the expression of epithelial cell adhesion molecules (E-cadherin, β-catenin and cytokeratins) is decreased, while the expression of mesenchymal proteins related to cell migration (vimentin, N-cadherin, etc.) is activated. Moreover, the participation of a network of transcription factors, including Snail, Slug, Twist and Zeb1, which repress the expression of cell adhesion molecules and trigger the manifestation of a mesenchymal phenotype, has been widely described [3,4]. It is noteworthy that EMT is not a binary process, since disseminated cancer cells can present intermediate phenotypes that are not completely epithelial or mesenchymal [3,5]. In addition, the EMT process may have other important implications for cancer cells such as the acquisition of stem cell-like properties [6,7,8], which are closely implicated in therapy resistance [9]. However, this is not always associated with EMT processes in the context of metastasis [1,10]. 

SOX9 is a member of the SOX family of developmental transcription factors, characterized by containing a high mobility group (HMG) DNA-binding protein domain. SOX9 plays a relevant role in development, governing cell fate specification and lineage commitment [11]. In the adult, SOX9 is involved in the regulation of homeostasis in several tissues including the pancreas, wherein it regulates stem cell maintenance and directs cell fate decisions in a dosage dependent manner [12,13]. Gain- and loss-of-function experiments have revealed that SOX9 activates EMT during embryonic development for neural crest delamination and cardiac valve formation [14,15], and also in different types of cancer [16,17,18]. Among them, it cooperates with members of the Snail/Slug family for metastatic progression in breast and lung cancers [8,19,20].

Pancreatic ductal adenocarcinoma (PDAC) is among the most lethal cancers, with a 5-year survival rate of less than 6% [21]. The poor prognosis of PDAC is mainly due to late diagnosis, spatial obstacles in resection, its high metastatic potential and its resistance to currently available chemotherapies [22,23,24]. The most effective therapeutic approach at present is surgical resection, yet most patients are diagnosed with an advanced stage disease and only a small proportion (around 15–20%) are eligible for surgery. Moreover, even in patients who receive surgical treatment, the majority of tumors will relapse [25]. Thus, unfortunately, pancreatic cancer still exhibits very close rates of incidence and mortality, with 458,918 new diagnoses and 432,242 deaths worldwide in 2018 [26], with metastasis being the leading cause of mortality. 

Several studies have demonstrated the involvement of SOX9 in the initial steps of pancreatic carcinogenesis. Thus, SOX9 is required for the formation of acinar-ductal metaplasias (ADM) and their progression to pancreatic intraepithelial neoplasias (PanIN), through its activity downstream of oncogenic K-RAS or EGFR signaling [27,28,29]; however, its role in the advanced stages of pancreatic cancer is not well described. Some evidence indicates that high SOX9 may promote pancreatic cancer cell invasion and aggressiveness [30,31,32], as well as chemoresistance [33], while others have reported low levels of SOX9 expression [28,34]. In this study we wanted to address whether SOX9 promotes EMT along with other metastatic traits in pancreatic cancer.

## 2. Experimental Procedures

### 2.1. Human Samples

Human samples arranged in tissue microarrays (TMAs) were provided by the Molecular Pathology Division of the Institute of Pathology at the Basel University Hospital. Written informed consent was obtained from all patients prior to specimen collection. 

### 2.2. Cell Culture

The pancreatic carcinoma cell lines Panc-1, RWP-1, IMIMPC-1, IMIMPC-2, BxPC-3, SKPC-1, SKPC-3, MiaPaca-2 and Hs766-T, were kindly provided by Dr. Real (CNIO) and Dr. Navarro (IMIM Medical Research Institute). All cell lines were mycoplasma free confirmed by the PCR-based detection of a conserved region of mycoplasma’s 16S rRNA using a mycoplasma detection kit (Biotools). Cells were cultured as adherent monolayers at 37 °C and 5% CO_2_ in a DMEM medium (Gibco) supplemented with 10% FBS (Gibco), 100 U/mL penicillin and 100 µg/mL streptomycin. Tumorspheres were cultured in DMEM/F12 medium (Sigma) supplemented with 20 ng/mL of EGF and bFGF (Sigma) growth factors, in the presence of N2 and B27. For the tumorsphere quantification studies, 1 × 10^3^ cells/well were seeded in non-treated 12-wells flat bottom plates and fresh media was added every 3 days. After 10 days, the 1ry (primary) tumorspheres were counted. Then, the spheres were disaggregated with accutase, seeded for 2ry (secondary) tumorspheres and maintained for another 10 days in culture. MEFs with a gain of SOX9 function have been described previously [35]. 

### 2.3. Gene Silencing and Overexpression

For SOX9 silencing by shRNA, cell lines were lentivirally infected. The cells were infected with lentivirus harboring the shSOX9 plasmid #40644 (*sh1*) and the corresponding pLKO.1 puro control plasmid #8453 (*pLKO*) from Addgene, both gifts from Dr. Bob Weinberg. For the lentiviral SOX9 overexpression, the Addgene plasmid #36979, a gift from Bob Weinberg, was used. For BMI1 upregulation, the cells were infected with lentivirus harboring the plasmid pLenti CMV GFP Puro-Bmi1 (a gift from Jacqueline Lees). Lentiviral infections were performed as previously described [36]. All infections were performed at a MOI of 10 for 6 h.

### 2.4. mRNA Expression Analysis

Total RNA was extracted with Trizol (Life Technologies, Carlsbad, CA, USA). Reverse transcription was performed using the High-Capacity cDNA Reverse Transcription Kit (ThermoFisher, Waltham, MA, USA) according to the manufacturer’s guidelines. Quantitative real-time PCR was performed in an ABI PRISM 7300 thermocycler (Applied Biosystems, Waltham, MA, USA) using a Power SYBR^®^ Green Master Mix (ThermoFisher), 10 mmol/L of primers and 20 ng of cDNA. *GAPDH*, a housekeeping gene, was used as a positive control for quantification. The ΔΔCT method was used for relative quantification. 

### 2.5. Western Blot and Immunofluorescence

Immunoblots and immunofluorescence were performed following standard procedures. Primary antibodies used were: SOX9 (AB5535, Millipore, Burlington, MA, USA), E-Cadherin (610181, BD Biosciences, San Jose, CA, USA), N-Cadherin (610920, BD Biosciences, San Jose, CA, USA), vimentin (M7020, Dako, Santa Clara, CA, USA), SNAIL (5243, AB clonal, Woburn, MA, USA), SLUG (bs-1382R, Bioss, Woburn, MA, USA) and β-actin (AC-15, Sigma, St. Louis, MO, USA).

### 2.6. In Vitro Assays for Migration and Invasion

For migration evaluation, wound healing (scratch) and transwell assays were performed. In the wound healing assays, the cells were seeded at a 90% confluency in 24-well flat bottom plates (triplicates) and 24 h later, a lineal artificial gap (scratch) was made with a sterile tip at the bottom of the wells. After removing the debris, the cells were serum-deprived for the duration of the experiment (48 h). Transwell migration was analyzed using Corning^®^ Transwell^®^ polycarbonate membrane inserts (#3422, Corning, NY, USA). Briefly, the cells were seeded into the insert compartment in medium without serum and the inserts were placed in wells of 24-well flat bottom plates containing medium with 10% of a fetal bovine serum as chemo-attractant. Then, 48 h later, the migrated cells were fixed and stained with 0.2% crystal violet in 5% formalin. To quantify the migration, the stain was dissolved and the absorbance was measured at 750 nm.

Invasion assays were performed using the QCM™ Collagen Cell Invasion Assay of Millipore (ECM551, Burlington, MA, USA). Invading cells were quantified 48 h after the seeding according to the manufacturer’s staining protocol.

### 2.7. F-Actin Staining

For cytoskeletal visualization, F-Actin was labelled with phalloidin. Cells were grown onto 8 mm diameter coverslips and fixed in 3.7% formaldehyde at room temperature for 15 min. After washing with PBS, the cells were permeabilized with 0.2% Triton X-100 (Sigma, St. Louis, MO, USA) and blocked with a blocking solution (10% *v/v* donkey serum in PBS/0.1% *v/v* Triton X-100; PBST) for 1 h. Then, F-actin filaments were stained using Alexa 546-conjugated phalloidin (Molecular Probes, Eugene, OR, USA) for 20 min at room temperature and the coverslips were counterstained with 4′,6-diamidino-2-phenylindole (DAPI) and mounted. Images were acquired using a Leica SPE confocal microscope. 

### 2.8. Colony Formation Assay

A total of 0.5 × 10^3^ cells were seeded in 9.5 cm^2^ wells (triplicates) in a DMEM medium. The medium was added every 3 days and after 10 days of culture the colonies formed were fixed with paraformaldehyde for 15 min at room temperature, stained with Giemsa and counted.

### 2.9. Soft Agar Foci

A total of 2.5 × 10^3^ cells were seeded in 9.5 cm^2^ wells (triplicates) in medium with 0.7% of agarose (top layer medium) on a bottom layer of medium with 1% of agar. The top layer medium was added every 3 days and after 15 days of culture the foci formed were counted. 

### 2.10. In Vivo Carcinogenesis Assays

For subcutaneous injection, cells were harvested with trypsin/EDTA and resuspended in PBS, with 1 × 10^5^ cells injected subcutaneously into both flanks of *Foxn1^nu^/Foxn1^nu^* nude mice (8 weeks old). External calipers were used to measure the tumor size at the indicated time points from which tumor volume was estimated according to the formula: ½ (length × width^2^). For tumor initiation experiments, 1 × 10^4^, 1 × 10^5^ and 1 × 10^6^ cells were injected into both flanks of *Foxn1^nu^/Foxn1^nu^* mice and tumor appearance was monitored over time.

For metastasis assays, *wt* and *Z/Sox9tg* MEFs and RWP1 and Panc-1 *pLKO/sh1* cells were injected intravenously into the tail vein of *Foxn1^nu^/Foxn1^nu^* mice. Metastatic *foci* were detected by in vivo imaging system and at endpoint mice were euthanized and *foci* were extirpated for pathologic and immunohistochemistry studies.

### 2.11. Immunohistochemistry

For immunohistochemistry, 4 micrometer-thick sections were incubated with primary antibodies: SOX9 (AB5535, Millipore, Burlington, MA, USA ), BMI1 (05-637, Millipore, Burlington, MA, USA), Ki67 (ab15580, abcam, Cambridge, UK), Snail (ab85931, abcam, Cambridge, UK), E-Cadherin (610181, BD Biosciences, San Jose, CA, USA), vimentin (M7020, Dako, Santa Clara, CA, USA), β-Catenin (610154, BD Biosciences, San Jose, CA, USA), and cytokeratin 19 (TROMA-III, Max Plant Institute, Freiburg, Germany). The sections then were washed and incubated with a MACH 3 Rabbit Probe and MACH 3 Rabbit HRP-Polymer (M3R531, Biocare Medical, Pacheco, CA, USA). The color was developed with 3,3′ diaminobenzidine (DAB, SPR-DAB-060, Spring Bioscience, Pleasanton, CA, USA). Counterstaining with hematoxylin was performed to mark the cell nuclei.

### 2.12. Expression Microarrays

HuGene 2.0_st arrays from Affymetrix (Santa Clara, CA, USA) were used to analyze the expression changes in RWP-1 cells with SOX9 silencing. The microarray data preprocessing was performed using the TAC software provided by Affymetrix including batch effect removal and the annotation file that corresponds to chip hugene_2_0_st_v1. The design and contrast matrices simply compared the shSOX9 with the references. Functional enrichment analysis was performed using the Overrepresentation Enrichment Analysis provided by WebGestalt, using a hypergeometric distribution to test the GO categories overrepresented. The enrichment analysis was performed independently for up- and down-regulated genes. Genes are included in the list if the fold change was larger than 1.5 (or less than −1.5 for down-regulated genes) and the *p* value was below 0.05. The selected universe for the enrichment analysis were the genes included in the hugene_2_0_st_v1 array. The functional analysis considered only the non-redundant biological processes terms. The data discussed in this publication have been deposited in NCBI’s Gene Expression Omnibus and are accessible in GSE193406.

### 2.13. Data Evaluation

Data are presented as mean values ± S.E.M. with the number of experiments (*n*) in parenthesis. Unless otherwise indicated, the statistical significance (*p*-values) was calculated using the Student’s t-test. The correlation *p* values related to the correlation coefficient (calculated using the Spearman method) have been calculated using the correlation test. Asterisks (*, **, and ***) indicate statistical significance (*p* < 0.05, *p* < 0.01, and *p* < 0.001, respectively).

## 3. Results

### 3.1. SOX9 Overexpression Confers Metastatic Potential and Promotes EMT in K-Ras Transformed Cells

**Figure 1:**
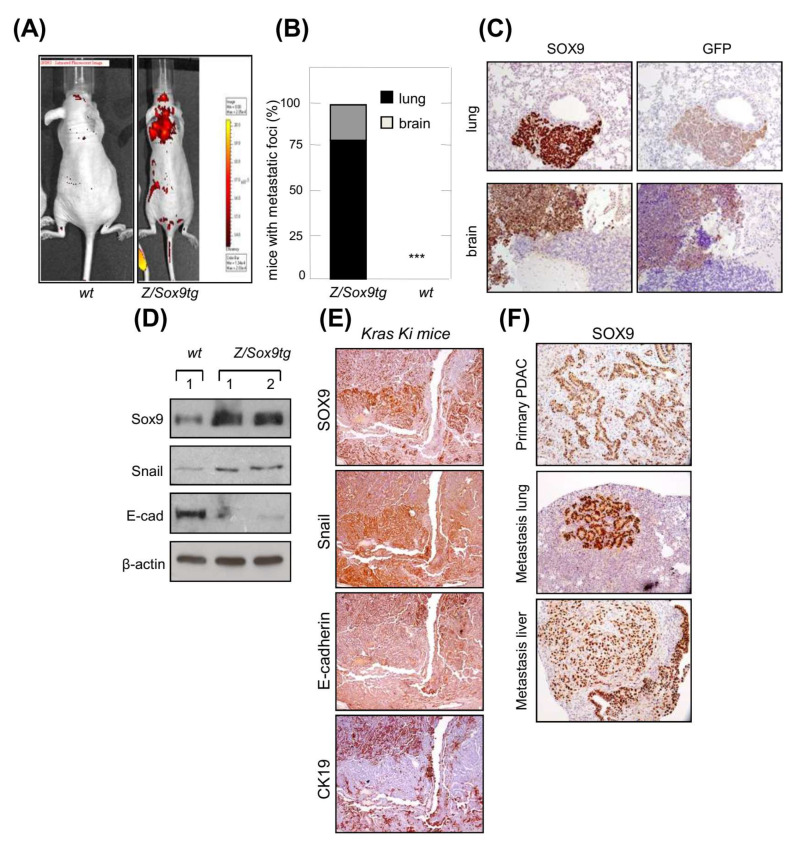
SOX9 overexpression promotes EMT and confers metastatic potential in *K-Ras* transformed cells. (**A**) Representative picture of metastatic tumors derived from intravenously injected *E1a/Ras* transformed MEFs with ectopic *Sox9* expression (*Z/Sox9tg*) (right, *n* = 5) or *wild type* MEFs (*wt*) (left, *n* = 5) in nude mice. Images obtained with an IVIS-200 in vivo imaging system. (**B**) Quantification of the percentage of nude mice that developed metastatic tumors derived from *Z/Sox9tg* (*n* = 5) and *wt* MEFs. (**C**) Sox9 and GFP expression analyzed by immunohistochemistry in lung and brain metastasis established by *E1a/Ras* transformed *Z/Sox9tg* MEFs. (**D**) Analysis by Western blot of Sox9, Snail and E-Cadherin levels in neoplastically transformed *wt* and *Z/Sox9tg* MEFs. (**E**) Serial sections of *K-Ras G12V*-induced lung adenocarcinomas stained with Sox9, Snail and the epithelial markers, E-cadherin and CK19. Representative image from at least three different mice. (**F**) Representative images of Sox9 expression analyzed by immunohistochemistry in sections from *K-Ras/p53^−/−^* induced primary pancreatic tumors and metastatic tumors in lung and liver (*n* = 5). *** indicates statistical significance with *p* < 0.001.

We examined whether SOX9 would facilitate the dissemination of cells to distant organs in vivo. For this, we used mouse embryo fibroblasts (MEFs), as they lack the ability to metastasize. *E1a/Ras* transformed MEFs that overexpress *Sox9* and *GFP* (*Z/Sox9tg*) and controls [35], were injected intravenously (tail vein) into nude mice. The progressive growth of these cells was traced in vivo and was detected in mice injected *with Z/Sox9tg* cells, but not in those injected with *wt* MEFs (Figure 1A). Consistent with these results, 100% of the *Z/Sox9tg* injected mice presented metastatic masses in the lungs or brain, whereas *wt* cells failed to establish metastases as judged by the absence of detectable tumors in these two organs or elsewhere (Figure 1B). The metastases found in the *Z/Sox9tg* injected mice were positive for SOX9 and GFP staining (Figure 1C), confirming that the tumors originated from *Z/Sox9tg* cells. Furthermore, along with the gain of metastatic potential, *Sox9* overexpression diminished the E-cadherin and increased Snail expression (Figure 1D). 

The most intensely SOX9 staining cells in the *K-Ras G12V*-induced lung primary adenocarcinomas were also the areas of tumor growth at invasive sites [35]. In order to expand these observations, we analyzed these areas for different EMT markers. The cells most strongly expressing SOX9, displayed intense staining for the EMT transcription factor Snail and an absence or low levels of E-cadherin and CK19 epithelial markers (Figure 1E). To further study the association between SOX9 and metastasis, we checked its expression in pancreatic *K-Ras(G12V);p53^−/−^* mice, which develop pancreatic tumors with a highly metastatic activity affecting lung and liver [37]. In this context, the staining of SOX9 was very intense in primary PDAC and in both lung and liver metastatic nodes (Figure 1F). 

### 3.2. SOX9 Overexpression Promotes EMT and Metastatic Traits in Pancreatic Cancer Cells

**Figure 2:**
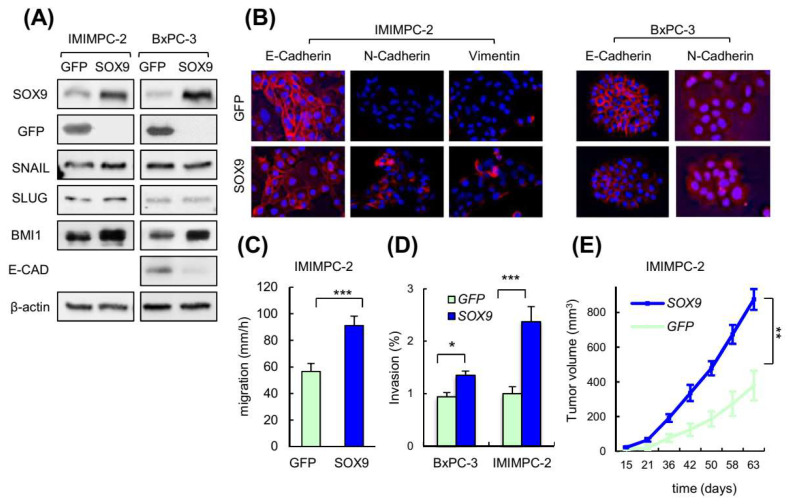
SOX9 overexpression promotes EMT and metastatic traits in pancreatic cancer cells. (**A**) Representative Western blot of GFP, SOX9, E-CAD, SNAIL, SLUG and BMI1 in IMIMPC-2 and BxPC-3 cell lines lentivirally transduced with plasmids harboring GFP or SOX9 coding sequences. (**B**) Representative images of epithelial (E-cadherin) and mesenchymal markers (N-cadherin and vimentin) expression detected by immunofluorescence in IMIMPC-2 and BxPC-3 cells transduced with GFP or SOX9 (*n* = 3). (**C**) Migration speed of IMIMPC-2 control and SOX9 cells calculated through live imaging studies (*n* ≥ 3). (**D**) Relative invasion of BxPC-3 and IMIMPC-2 cell lines with ectopic SOX9 overexpression with respect to the invasion of cells transduced with GFP. Invasive cells analyzed using collagen transwell assays based in Boyden chamber (*n* = 3). (**E**) Tumor volume over time of subcutaneous tumors formed in nude mice by control and SOX9 transduced IMIMPC-2 cells (*n* = 12). Asterisks (*, **, and ***) indicate statistical significance (*p* < 0.05, *p* < 0.01, and *p* < 0.001, respectively).

We tested whether high levels of SOX9 were sufficient to promote EMT in pancreatic cancer cells, which are classified as the tumor cell types with high SOX9 expression (Supplementary Figure S1A). To address this, we transduced primary epithelial BxPC-3 and IMIMPC-2 PDAC cell lines, which express endogenous low levels of SOX9 (Supplementary Figure S1B,C), with plasmids encoding *SOX9* or GFP. Immunoblotting revealed an overexpression of SOX9 in both cell lines (Figure 2A). In this context, SOX9 overexpression was associated with decreased E-cadherin along with higher vimentin and N-cadherin expression (Figure 2A,B), reflecting the activation of EMT. In agreement, SOX9 overexpressing cells presented a less cohesive organization when compared to control cells (Supplementary Figure S2A). Surprisingly, SOX9 overexpression did not alter the expression of SNAIL or other well-known EMT transcription factors such as SLUG or ZEB1 in BxPC-3 and IMIMPC-2 pancreatic cancer cells (Figure 2A; Supplementary Figure S2B); however, the expression of the BMI1 stem cell factor was elevated (Figure 2A).

As cancer cell motility and invasion into the basement membrane are associated with the induction of EMT and metastasis [6], we investigated these capabilities in pancreatic cancer cells overexpressing SOX9. For this, we performed scratch assays followed by live-cell microscopy, finding that PDAC cells with SOX9 overexpression displayed significantly enhanced migratory potential (Figure 2C; Supplementary Videos S1 and S2). Moreover, transwell assays also demonstrated that high levels of SOX9 boost invasion into collagen (Figure 2D). Successful colonization of distant organs requires active and highly proliferative cells to generate micrometastasis and macrometastasis [2,38]. We recently showed that ectopic SOX9 upregulation increased the proliferation of BxPC-3 and IMIMPC-2 cells [39]. This effect was paralleled by enhanced tumor growth over time in vivo (Figure 2E). Altogether, these observations establish that high levels of SOX9 in pancreatic cancer cells facilitate their acquisition of the cellular and molecular traits required for the metastatic dissemination and colonization.

### 3.3. Silencing of SOX9 Drives MET and Decreases Metastatic Potential

**Figure 3:**
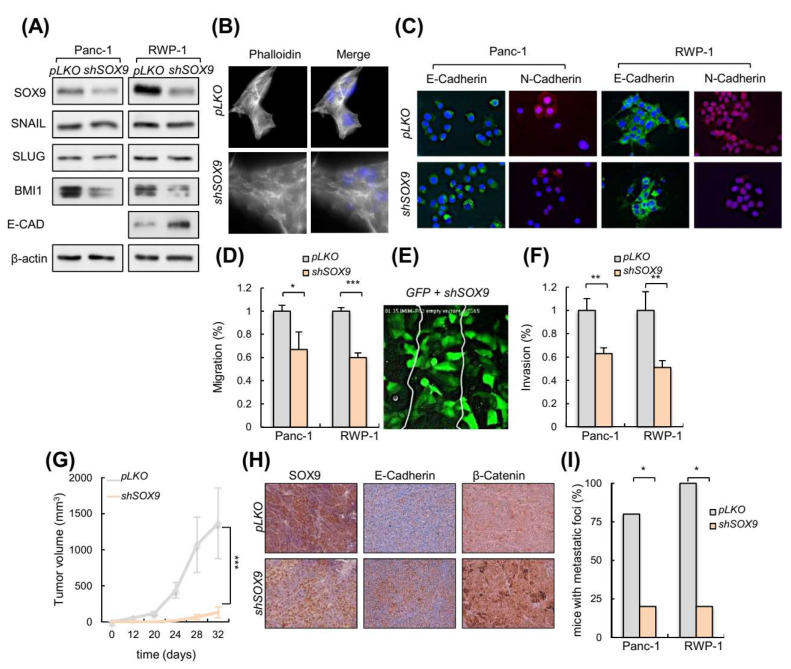
SOX9 knockdown drives MET and impairs metastasis. (**A**) Representative Western blot of SOX9, E-CAD, SNAIL, SLUG and BMI1 protein levels in Panc-1 and RWP-1 cell lines lentivirally infected with virus harboring a specific short hairpin RNA against SOX9 (*shSOX9*) or the corresponding empty vector (*pLKO*) (*n* ≥ 4). (**B**) Images show filamentous actin (F-actin) labeled with phalloidin in RWP-1 *pLKO* and *shSOX9* cells. Nuclei were marked with DAPI, and the merged images are presented in right panel. (**C**) Representative images of E-Cadherin and N-Cadherin expression determined by immunofluorescence in Panc-1 and RWP-1 *pLKO* and *shSOX9* cells (*n* = 3). (**D**) Relative migration determined by transwell assays based in Boyden chamber (*n* ≥ 4) in indicated cells and genotypes. (**E**) Frame at the time of wound closure (from Supplementary Video S2: down, middle panel) obtained in a scratch assay performed with a mix of IMIMPC-2 cells transduced with *GFP* or *shSOX9*. (**F**) Relative invasion of Panc-1 and RWP-1 cell lines with *shSOX9* with respect to *pLKO* cells. Invasive cells analyzed in vitro using collagen transwell assays based in Boyden chamber. (**G**) Tumor volume at the indicated time points of subcutaneous tumors formed by control *pLKO* and *shSOX9* RWP-1 cells (*n* = 12). (**H**) Representative images of SOX9, E-Cadherin and β-Catenin expression determined by immunohistochemistry in subcutaneous tumors represented in 3G. (**I**) Percentage of nude mice that developed metastatic *foci* derived from Panc-1 and RWP-1 *pLKO* and *shSOX9* cells in tail vein injection assays (*n* = 5). Asterisks (*, **, and ***) indicate statistical significance (*p* < 0.05, *p* < 0.01, and *p* < 0.001, respectively).

We then studied whether SOX9 knockdown could impair metastatic traits. For this, we silenced SOX9 in Panc-1 and RWP-1, two highly invasive and metastatic human pancreatic cancer cell lines, both with elevated endogenous levels of SOX9 (Supplementary Figure S1B). Western blotting confirmed the knockdown of SOX9 in both cell lines (Figure 3A). Phase contrast images and F-actin staining with phalloidin showed that cells with silenced SOX9 acquired an epithelial-like morphology (Figure 3B; Supplementary Figure S3A). According to this, SOX9 silencing increased the E-cadherin levels and reduced N-cadherin levels (Figure 3A,C), resembling the process of MET; however, the expression of *SNAIL*, *SLUG, ZEB1* or *PRRX1* was not altered in the SOX9-silenced cells, whereas BMI1 levels were reduced (Figure 3A; Supplementary Figure S3B). These results, together with the data presented above from the SOX9 overexpressing cells, suggest that EMT transcription factors are not linked to SOX9 functions in pancreatic cancer. Supporting this idea, their expression correlated negatively with SOX9 expression in human pancreatic cancer samples from the TCGA cohort (Supplementary Figure S3C).

We performed scratch assays with live-cell microscopy in pancreatic cancer cells with and without SOX9 silencing. Live-cell microscopy compositions from these assays revealed an impaired migration in SOX9 knockdown cells (Figure 3D; Supplementary Videos S2 and S3), which were able to form protrusive structures at the leading edge but seemed not to achieve the traction necessary to move efficiently forward. To give further insight into which cells are most efficient at wound healing in a competence context, we co-cultured IMIM-PC2 cells transduced with GFP and shSOX9 and we observed that the scratch was mainly closed by GFP transduced cells (Figure 3E, Supplementary Video S2). Accordingly, SOX9-silenced cells were found to invade the collagen matrix assays less efficiently than control cells (Figure 3F).

We performed in vivo studies in immunodeficient mice. When a subcutaneous inoculation route was used, tumors derived from *SOX9*-silenced cells exhibited a markedly reduced growth with respect to the tumors derived from control cells (Figure 3G). Of note, tumors derived from *SOX9*-silenced cells presented higher E-cadherin and β-catenin expression (Figure 3H), further supporting the link between SOX9 and the EMT process. Then, we compared the ability of control and *shSOX9* transduced cells to form metastatic *foci* in the lungs after intravenous injection in the tail vein. These experiments revealed a reduced metastatic potential in *SOX9*-silenced cells, with less than 20% of mice injected with *shSOX9* presenting *foci*, compared to almost 100% of the mice with controls that developed metastatic *foci* in their lungs (Figure 3I). These results altogether, support the notion that SOX9 activity is necessary for pancreatic cancer dissemination and colonization.

### 3.4. SOX9 Is Necessary for Pro-Metastatic Cancer Self-Renewal

**Figure 4:**
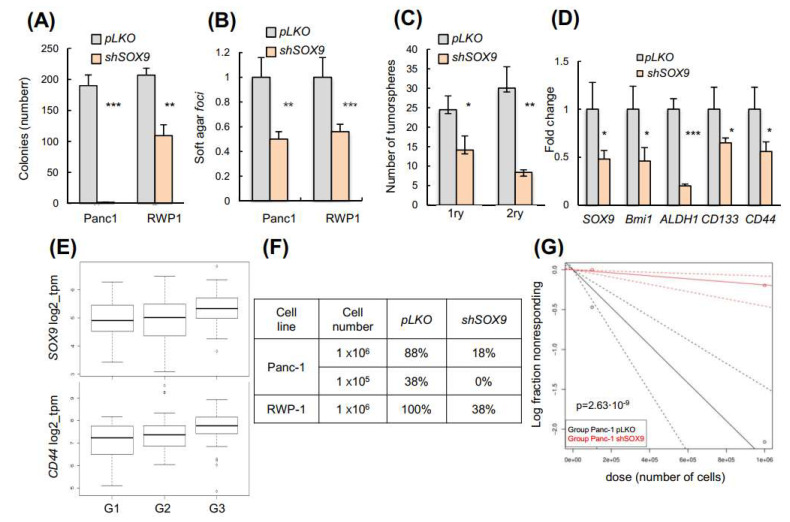
SOX9 is necessary for pro-metastatic cancer self-renewal. (**A**) Number of colonies formed by Panc-1 and RWP-1 *pLKO* and *sh1* (*n* ≥ 4). (**B**) Relative number of soft agar foci formed by Panc-1 and RWP-1 *sh1* cells (*n* = 4). (**C**) Representative image and quantification of the number of primary (1ry) and secondary (2ry) tumorspheres formed by control and *sh1* RWP-1 cells (*n* = 6). (**D**) mRNA levels of cancer stem cell markers in 2ry tumorspheres (*n* = 4). (**E**) SOX9 and CD44 expression according to tumor grade in pancreatic cancer samples from the TCGA cohort (SOX9: *p* = 0.0289, *n* = 147; CD44: *p* = 0.0038, *n* = 147). (**F**) Tumor incidence in nude mice injected with Panc-1 and RWP-1 *pLKO* and *sh1* cells. (**G**) Plot representing the fraction of mice without tumors with respect to the dose of Panc1 cells subcutaneously injected. The slopes of the depicted solid lines correspond to the fraction of cells with tumor-initiating ability (black color: *pLKO* cells; red color: *sh1* cells. Chi square test for differences in stem cell frequencies: *p* = 2.63 × 10^−9^. Analysis performed using the ELDA software application (http://bioinf.wehi.edu.au/software/elda/). Accessed on 6 September 2021. Asterisks (*, **, and ***) indicate statistical significance (*p* < 0.05, *p* < 0.01, and *p* < 0.001, respectively).

We evaluated whether SOX9 might function in the maintenance of CSCs. We carried-out colony formation, soft agar and tumorsphere formation assays to examine the ability of cells to grow independently of attachment and their self-renewal potential in vitro. Silencing of SOX9 in the RWP-1 and Panc-1 cells resulted in a significant decline in the number of colonies and *foci* in soft agar (Figure 4A,B), and reduced the tumorsphere formation ability in stem-cell selective media (Figure 4C). To examine this further we analyzed the expression of the well-established pancreatic CSCs markers, *CD133*, *CD44*, *BMI1*, and *ALDH1* (39), which were significantly lower in *shSOX9* tumorspheres (Figure 4D). Consistent with these observations, SOX9 expression positively correlated with *CD44* and *BMI1* in the TCGA cohort samples (Supplementary Figure S3C). Moreover, the expression of *CD44* and *SOX9* increased according to tumor grade in the samples of patients (Figure 4E).

We determined the effect of *SOX9*-silencing on tumor initiation by performing subcutaneous injections upon limiting dilution transplantation in immunocompromised mice in vivo. We found that *SOX9*-silencing in both Panc-1 and RWP-1 cells resulted in a significant impairment of their tumorigenic ability (Figure 4F). The analysis using the ELDA software application revealed a diminished frequency of tumor-initiating/cancer stem cells in SOX9-silenced cells, with frequencies of 1/422,319 and 1/5,255,599 for the control and *shSOX9* cells, respectively (Figure 4G). Altogether, these results confirm that SOX9 sustains the self-renewal and tumor initiation activity in pancreatic cancer.

### 3.5. Transcriptomics Reveal Multiple Processes Differentially Expressed in SOX9-Silenced Cells

**Figure 5:**
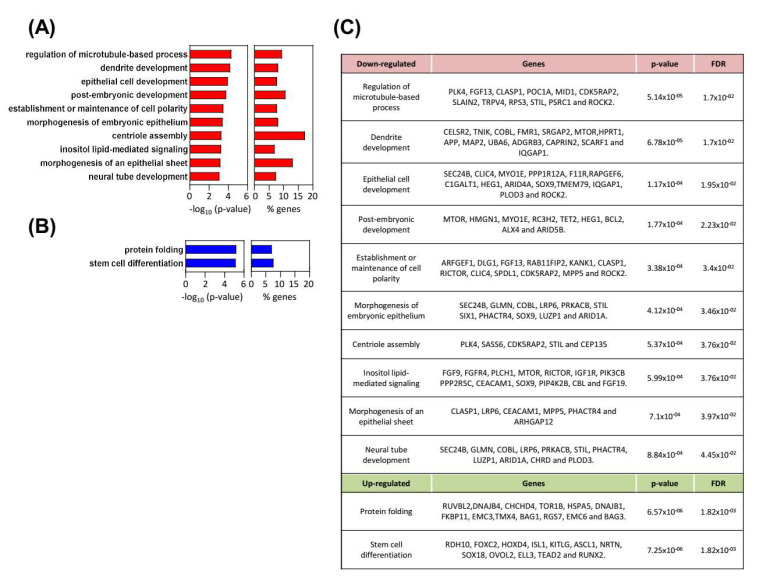
Multiple biological processes differentially expressed in *SOX9*-silenced cells. (**A**,**B**) Biological processes (BPs) altered in *shSOX9* RWP-1 cells. Gene ontology enrichment analysis from the differentially expressed genes were performed with WebGestalt (WEB-based Gene SeT AnaLysis Toolkit). Bar charts represent −log10 of the *p*-values and the percentage of genes belonging to the different BPs whose expression is altered. BPs (**A**) downregulated are represented in red and (**B**) upregulated in blue. (**C**) Differentially expressed genes in *SOX9*-silenced cells belonging to the identified BPs.

We performed expression microarrays in RWP-1 *pLKO* and *shSOX9* cells. An amount of 1091 genes were differentially expressed between conditions with *p* values below 0.05 and a fold change higher than 1.5 (or less than −1.5 for down-regulated genes). Functional enrichment analysis developed using the Overrepresentation Enrichment Analysis provided by WebGestalt and a hypergeometric distribution to test the GO categories overrepresented, revealed an alteration of different biological processes in agreement with the cellular features observed with SOX9 modulation. Thus, the establishment or maintenance of cell polarity, and the centriole assembly or regulation of microtubule-based processes, which are related to motility and migration, were downregulated in *SOX9*-silenced cells (Figure 5A). The two latter processes were also important in cytokinesis and cell division, which is concordant with the less proliferative phenotype exhibited by *SOX9*-silenced cells. Moreover, downregulation of neural tube development and post-embryonic development were detected in *SOX9*-silenced cells (Figure 5A). On the contrary, the stem cell differentiation processes were upregulated with *SOX9*-silencing (Figure 5B), which is in agreement with the SOX9 function in stem cell activity. Among the list of genes differentially expressed, genes involved in EMT and metastasis including *FGFR4*, *mTOR-RICTOR*, *PLK4*, and *PIK3CB* were found; however, EMT factors were not detected (Figure 5C). This result further supports that SOX9 controls different EMT states in pancreatic cancer cells.

### 3.6. BMI1 Stem Cell Factor Mediates SOX9 Activities

**Figure 6:**
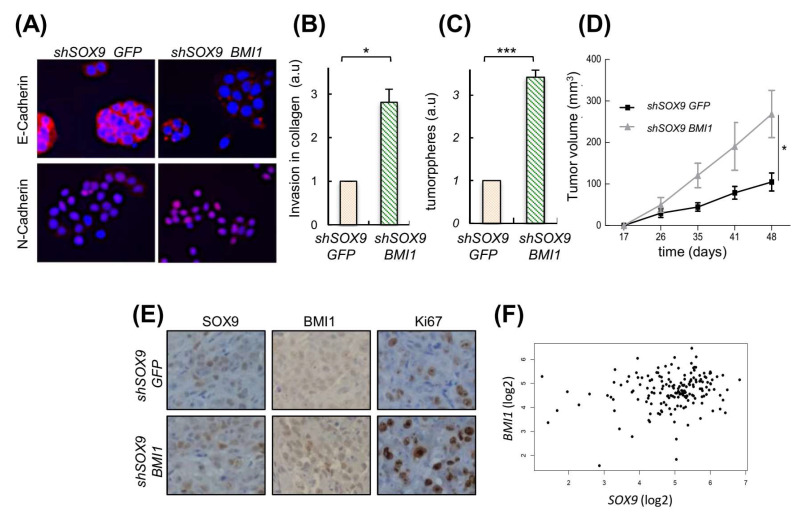
BMI1 is relevant in the metastatic potential induced by SOX9. (**A**) E-Cadherin and N-Cadherin expression analyzed by immunofluorescence in *SOX9*-silenced RWP-1 cells transduced with BMI1 (*shSOX9 BMI1*), and *SOX9*-silenced RWP-1 cells transduced with the corresponding empty plasmid (*shSOX9 GFP*). (**B**) Relative invasion determined by transwell assays for *shSOX9 BMI1* referred to *shSOX9GFP* (*n* = 4). (**C**) Relative number of tumorspheres (*n* = 3). (**D**) Average volume at the indicated time points of tumors derived from Panc-1 *shSOX9 BMI1* and *GFP* (10 × 10^4^ cells/injection). (**E**) Representative images of SOX9, BMI1 and Ki67 expression determined by immunohistochemistry in subcutaneous tumors represented in 6D. (**F**) Correlation between the expression of SOX9 and BMI1 in pancreatic tumors of the TCGA cohort (cor = 0.19; *p* = 0.0137). * and *** indicate statistical significance with *p* < 0.05 and *p* < 0.001, respectively.

We have previously found that the transcriptional repressor BMI1, which is an important regulator of self-renewal, linked to EMT and metastasis in different cancers [40,41] including PDAC [42,43], is an effector of SOX9 activity [39]. We tested whether it could be involved in SOX9-mediated EMT and self-renewal activity. For this, we ectopically re-expressed *BMI1* in *SOX9*-silenced cells. Interestingly, ectopic *BMI1* restoration rescued the expression of N-cadherin and diminished the expression of E-cadherin (Figure 6A). In agreement with this, cells with *SOX9*-silencing and *BMI1* restoration exhibited a 3-fold increase in their invasive potential compared to cells with *SOX9*-silencing (Figure 6B). Moreover, the *BMI1* restoration increased in 3.4-fold the tumorsphere formation capacity of cells (Figure 6C). Accordingly, *BMI1* significantly fostered in vivo tumor growth (Figure 6D), as well as the number of Ki67 positive cells in vivo (Figure 6E). The link between SOX9 and BMI1 was translated to clinical samples since their levels positively correlated in PDACs from the TCGA cohort (Figure 6F).

### 3.7. High Levels of SOX9 Confer Chemoresistance

**Figure 7:**
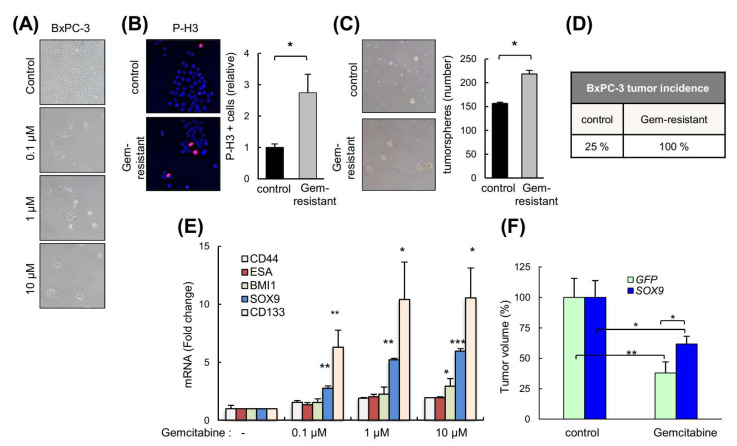
High levels of SOX9 confer chemoresistance. (**A**) Representative images of BxPC-3 cells untreated (parental) and gemcitabine-resistant cells after 120 h of treatment with different concentrations of gemcitabine (0.1, 1 and 10 µM). (**B**) Representative image and representation of the proportion of BxPC-3 and BxPC-3 gemcitabine-resistant cells (Gem-resistant) positive for the mitosis marker phospho-histone H3 (*p*-H3). (**C**) Representative image and quantification of the number of tumorspheres derived from BxPC-3 and BxPC-3 gemcitabine-resistant cells. (**D**) Percentage of subcutaneous tumors formed from BxPC-3 parental and gemcitabine-resistant cells (1 × 10^6^ cells/injection). (**E**) *SOX9*, *CD133*, *BMI1*, *CD44* and *ESA* mRNA expression in BxPC-3 control and gemcitabine-resistant cells (*n* = 4). (**F**) Volume of tumors from IMIM-PC2 *GFP* and SOX9 cells in nude mice treated intraperitoneally once per week with vehicle or gemcitabine 5 mg/Kg (*n* = 5). Asterisks (*, **, and ***) indicate statistical significance (*p* < 0.05, *p* < 0.01, and *p* < 0.001, respectively).

Since our data indicated that SOX9 induces EMT and stemness, we reasoned that SOX9 might be involved in resistance to chemotherapy. Therefore, we exposed BxPC-3 cells to cytotoxic doses of gemcitabine in order to obtain drug-resistant cells (Figure 7A). These resistant cells were highly proliferative (Figure 7B), formed a higher number of tumorspheres (Figure 7C), and presented higher tumor initiation activity than the parental cells (Figure 7D). Gemcitabine-resistant cells exhibited a significantly elevated *SOX9* expression 5.2- and 6-fold in 1 μM and 10 μM concentrations, respectively, as well as increased levels of stem cell markers such as *CD133*, *BMI1*, *CD44* and *ESA* (Figure 7E). Moreover, the reduction in tumor volume exerted by the gemcitabine was significantly lower in tumors overexpressing SOX9 (Figure 7F). These results support the notion that SOX9 confers gemcitabine chemoresistance in pancreatic cancer.

### 3.8. High Levels of SOX9 Are Associated with Reduced Survival and Correlate with EMT Markers

**Figure 8:**
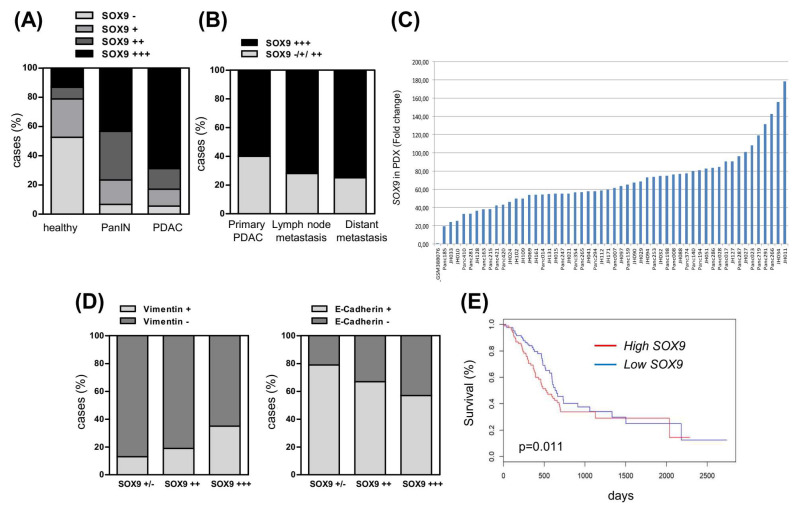
High levels of SOX9 are associated with PDAC progression and reduced survival. (**A**) SOX9 intensity analyzed by immunohistochemistry in a tissue microarray (TMA) composed by human samples of healthy pancreas (*n* = 38), PanIN (*n* = 30) and PDAC (*n* = 198). (−): 0–20 intensity grade; (+): 30–50 intensity grade; (++): 60–80 intensity grade; (+++): 90–100 intensity grade. (**B**) Distribution of SOX9 expression analyzed by immunochemistry in PDAC samples according to stage. Primary PDAC (*n* = 58), lymph node metastases (*n* = 123) and distant metastases (*n* = 12). (**C**) SOX9 mRNA expression in 53 pancreatic tumors from patient-derived xenografts (PDXs) relative to the average SOX9 expression level found in 36 samples of normal human pancreas. Expression in normal tissue is included as first line (GSM388076), whereas the remaining lines are the independent PDXs with the respective number in the bottom. (**D**) Negative association between SOX9 and vimentin protein expression (chi square, *p* = 0.0006) and positive association between SOX9 and E-Cadherin expression (chi square, *p* = 0.003) in human PDAC. Data obtained by immunohistochemistry in the TMA of 198 human PDAC samples. (**E**) Overall survival analysis of pancreatic cancer patients from the TCGA cohort according to SOX9 expression (*p* = 0.011, *n* = 178).

We investigated SOX9 expression in a large number of samples of healthy human pancreatic tissue, and in PanIN and PDAC by immunohistochemistry on a tissue microarray (TMA) [44]. SOX9 was expressed, although generally at low levels, in healthy pancreatic tissue, while its expression increased in PanIN lesions and was highest in PDAC (Figure 8A). Interestingly, metastatic samples (lymph nodes and distant organs) displayed very high SOX9 expression (^+++^) (Figure 8B). Consistent with this, the levels of SOX9 were higher in metastatic cell lines such as IMIMPC-1, RWP-1, Hs766T, and the highly pro-metastatic cell line Panc-1, than in the primary pancreatic cancer cell lines (Supplementary Figure S1B). Patient-derived xenografts (PDXs) represent to a certain extent the original human pancreatic tumors [45]. The distribution of SOX9 in a set of 53 engrafted tumors was reminiscent of patient data, with all of them displaying higher SOX9 mRNA expression (from 20- to 178-fold) compared to the average expression found in a group of 36 normal human pancreatic tissue samples (Figure 8C).

Next, we investigated whether SOX9 expression was associated with EMT markers. For this, we analyzed the expression of E-cadherin (epithelial) and vimentin (mesenchymal) on the TMA. Consistent with previous studies [46,47], we observed high vimentin expression (≥10% of cells) coupled with a total or partial loss of E-cadherin (≤90% of cells) in PDAC (data not shown). Interestingly, the correlation analysis showed a positive association between SOX9 and vimentin expression (chi square, *p* = 0.0006) (Figure 8D). Alongside, there was an inverse correlation between SOX9 and E-cadherin expression (chi square, *p* = 0.003) (Figure 8D). Moreover, the analysis of the available information from The Cancer Genome Atlas (TCGA) project associated high SOX9 expression with reduced overall survival (*p* = 0.011) (Figure 8E), showing the relevance of SOX9 in the clinical progression of pancreatic cancer.

## 4. Discussion

Metastasis is the main cause of cancer deaths and remains the least understood process of cancer progression. This challenge is particularly important in pancreatic cancer, whereby the metastatic dissemination of cells occurs even at premalignant stages [48]. In this work, we demonstrate that SOX9 is necessary and sufficient for the activation and modulation of a tumorigenic EMT in pancreatic cancer, in a process that requires the activation of stemness pathways, but not critical EMT factors. Moreover, we ascertain that high levels of SOX9 are associated with poor patient survival and chemoresistance to gemcitabine.

We confirmed in clinical samples that SOX9 expression was particularly high in samples from metastases in both lymph nodes and distant organs. Importantly, through both gain- and loss-of-function approaches, we functionally demonstrated that SOX9 promotes the migration and invasion of pancreatic cancer cells and facilitates the metastatic dissemination and colonization of distant organs in vivo. Consistent with these observations, several studies have linked SOX9 activity to invasion and metastasis in pancreatic cancer [31,32,49] and additional types of cancer [20,50,51,52,53]. Our results are in agreement with the findings revealing that SOX9 activates context-dependent tumorigenic EMT programs [20,54,55,56]. Indeed, both gain- and loss-of-function approaches revealed that its activity is necessary for the induction of EMT and MET in *K-Ras* transformed cells and pancreatic tumor cells. Moreover, SOX9 correlated negatively with E-cadherin (epithelial marker) and positively with vimentin (mesenchymal marker) in human PDAC samples and pancreatic cells; however, the activation of EMT seems to be independent of SNAIL/SLUG or additional EMT transcription factors such as ZEB1 or PRRX1 in the advanced stages, because these transcription factors are not affected in pancreatic cancer cells with an altered expression of SOX9. Thus, our results show that SOX9 acts similarly to other EMT inducers including SNAIL, SLUG and ZEB1, which induce EMT along with stemness [6,7,8], and in contrast to PRRX1, which governs a pro-tumorigenic EMT not linked to stem cell properties [1,10].

Our results also support the notion that metastasis is in most cases dependent on EMT processes, which could be partial and are necessary for primary tumor cells to become motile and invasive [57]. Indeed, particularly in pancreatic cancer, a study using a mouse model with pancreas-specific alteration of pancreatic cancer driver genes, that harbored a lineage system to track the cells of epithelial origin, revealed that cancer circulating cells expressing mesenchymal markers were able to seed the liver [58]. In contrast, another study also using genetically engineered mouse models affirmed that EMT is dispensable for pancreatic cancer metastasis, but it is essential for therapy resistance [59], a phenomenon also observed in lung cancers [60]. This study showed that EMT specifically driven by Snail or Twist was not necessary for pancreatic cancer metastasis.

Our results also demonstrate that SOX9 confers resistance to chemotherapy in PDAC. In accordance with other studies, we observed an enrichment of the CSC compartment after gemcitabine exposure [61,62,63,64]. Importantly, SOX9 expression was elevated in gemcitabine resistant cells to a level only reached by *CD133*, one of the best-established stem cell markers in pancreatic cancer [62]. Additional studies also reported an increase of SOX9, although one of them of smaller magnitude, with lower concentrations of gemcitabine [30,33], suggesting a dose dependent effect of this agent. Taken together, these data link SOX9 to pancreatic CSCs and chemoresistance. In addition, our results further support the link between EMT and chemoresistance observed in pancreatic cancer [59,65]. Finally, we found that high levels of SOX9 correlated with poor patient survival. These results are in line with previous studies [33,66] and support the idea that SOX9 is a prognostic biomarker of pancreatic cancer.

## 5. Conclusions

In summary, we demonstrate that the developmental transcription factor, SOX9, confers cellular plasticity and promotes EMT in pancreatic cancer cells. This facilitates metastasis and promotes resistance to therapies, being both directly responsible for disease relapse and death. At the cellular level, SOX9 is a pleiotropic regulator of cancer cell activity governing additional functions to its well-known function regulating quiescence and self-renewal, such as EMT and metastasis. Mechanistically, we have identified stem cell factors rather than EMT factors as important mediators of the oncogenic activity of SOX9 in pancreatic cancer. Altogether, our findings highlight the relevance of SOX9 in pancreatic cancer outcomes due to its role in metastasis and to recurrence after therapy.

## Data Availability

The data that support the findings of this study are included in the manuscript or in Supplementary Figures. Microarray data are openly available in GEO database with access number GSE193406.

## Supplementary Materials

The following supporting information can be downloaded at: https://www.mdpi.com/article/10.3390/cancers14040916/s1, Figure S1: High levels of SOX9 in pancreatic cancer cells, Figure S2: Characteristics of SOX9 overexpressing cells, Figure S3: Characteristics of SOX9 knock-down cells; Video S1: Frame composition showing motility and migration of IMIMPC-2 control (GFP) and SOX9 up-regulated (SOX9) cells in an in vitro scratch (wound healing) assay. Composition made from images acquired with a live cell imaging system over night, Video S2: Frame composition showing motility and migration performed by IMIMPC-2 cells with ectopic SOX9 overexpression or silencing in scratch (wound healing) assays. Up: Left panel: control cells (IMIM empty vector). Middle panel: IMIMPC-2 cells with ectopic SOX9 overexpression (IMIM OE SOX9). Right panel: SOX9-silencing cells (IMIM shSOX9). Down: Left panel: mix of IMIMPC-2 control cells harboring the empty vector with GFP (green cells) and IMIM-PC2 cells with ectopic SOX9 overexpression (IMIM empty vector+ IMIM OE SOX9). Middle panel: mix of IMIM-PC2 control cells harboring the empty vector with GFP (green cells) and IMIMPC-2 cells with SOX9-silencing (IMIM empty vector+ IMIM shSOX9), Video S3: Frame composition showing motility and migration performed by RWP-1 control (pLKO) and RWP-1 SOX9-silenced cells (sh1) in an in vitro scratch (wound healing) assay. Composition made from images acquired with a live cell imaging system over night.
