# Supplementary material for: SOX9 Triggers Different Epithelial to Mesenchymal Transition States to Promote Pancreatic Cancer Progression
Source: Cancers (Basel). 2022 Feb 12;14(4):916. doi: 10.3390/cancers14040916 (PMC8870732; doi:10.3390/cancers14040916)
Supplement: Supplementary file 1 [file cancers-14-00916-s001.zip › cancers-1571218-supplementary/cancers-1571218-supplementary.pdf]

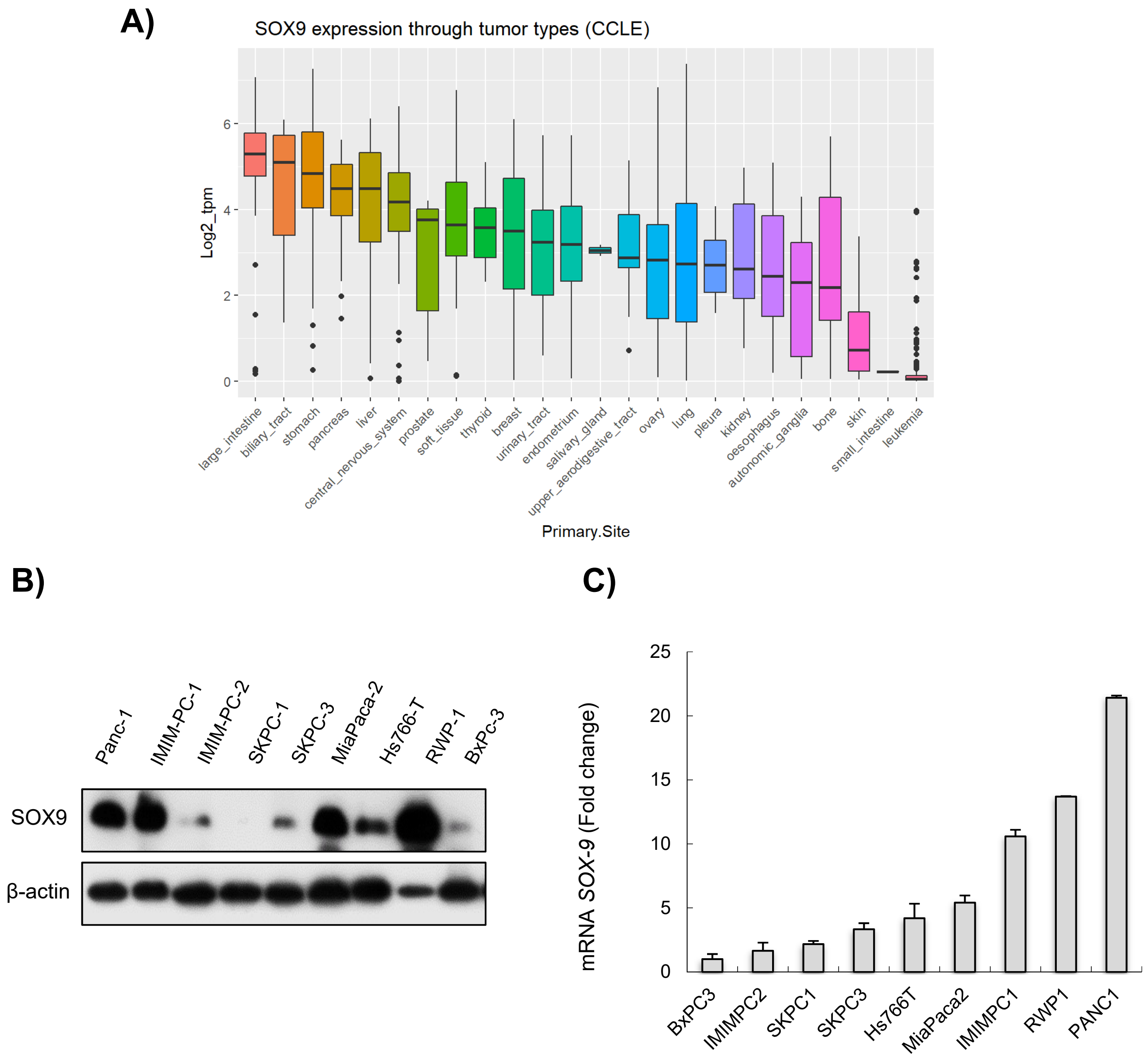

Suppl. Figure S1: High levels of SOX9 in pancreatic cancer cells

(**A**) Box plots representing *SOX9* expression in cancer cell lines grouped according to the primary tumor site. *SOX9* expression in pancreatic tumors ranks fourth out of a total of 24 different tissues. Information extracted from The Cancer Cell Line Encyclopedia (CCLE) (www.broadinstitute.org/ccle). (**B**) Representative Western blot of SOX9 in a panel of pancreatic cancer cell lines. Metastatic cell lines: RWP-1 and IMIMPC-1 (liver metastasis), and Hs766T (lymph node metastasis). Poor differentiated cell lines: Panc-1, SKPC-3 and MiaPaca-2. More differentiated cell lines: IMIMPC-2, SKPC-1 and BxPC-3. (**C**) Representation of *SOX9* mRNA expression the panel of 9 pancreatic cancer cell lines represented in Supp 1B.

A)

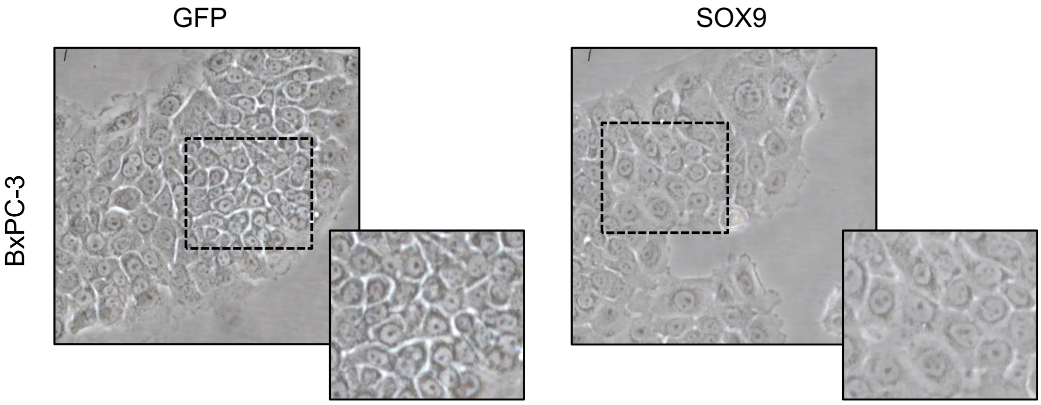

B)

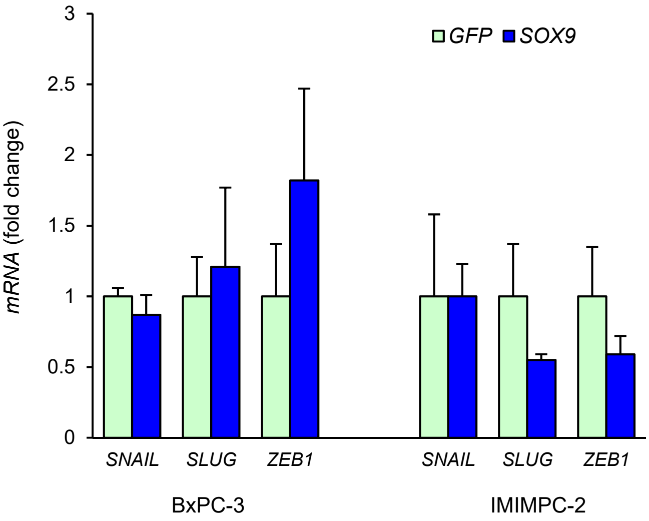

Suppl Figure S2: Characteristics of SOX9 overexpressing cells.

(**A**) Representative image showing the morphology and organization of *SOX9* up-regulated BxPC-3 cells (SOX9) and BxPC-3 cells transduced with the corresponding empty vector (GFP). (**B**) *SNAIL, SLUG* and *ZEB1* mRNA expression in primary PDAC cell lines with ectopic *SOX9* overexpression (n≥3).

A)

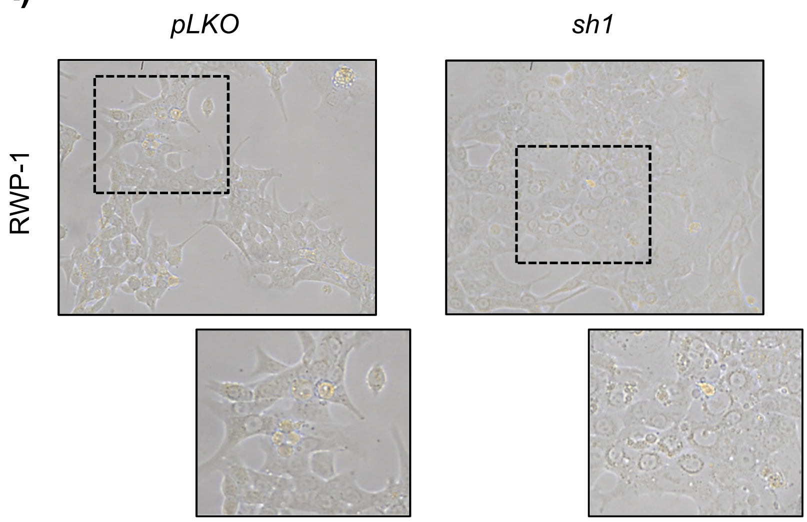

B)

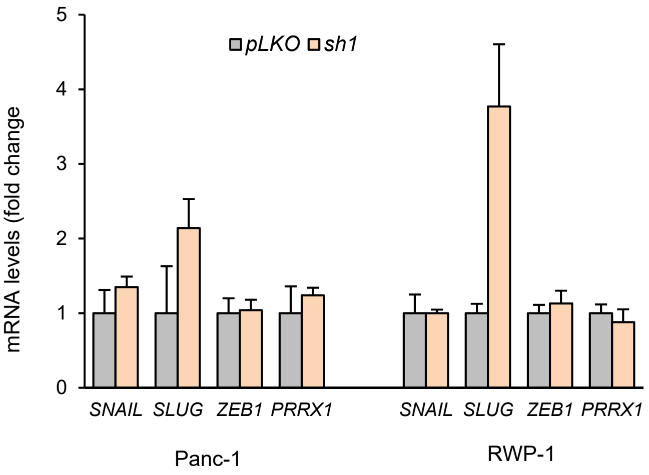

C)

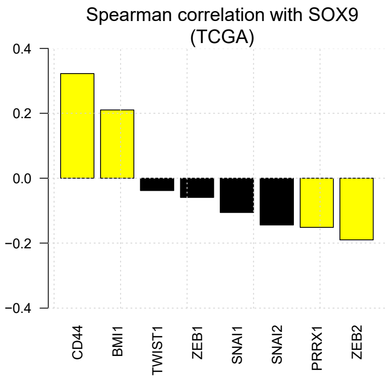

Figure Suppl S3: Characteristics of SOX9 knock-down cells.

(**A**) Representative images showing the epithelial morphology acquired by RWP-1 cells in response to SOX9 silencing (sh1). (**B**) *SNAIL, SLUG, ZEB1* and *PRRX1* mRNA expression in *SOX9*-silenced PDAC cells (sh1) respect to control cells transduced with the empty vector (pLKO) (n≥3). (**C**) Spearman correlation of the expression of the indicated genes with *SOX9* expression in the samples of pancreatic cancer of TCGA. Significant in yellow (p≤0.05).
